# P028. Childhood migraine, epilepsy and tics: Are there similarities in the psychological profile?

**DOI:** 10.1186/1129-2377-16-S1-A152

**Published:** 2015-09-28

**Authors:** Samuela Tarantino, Simona Cappelletti, Maria Francesca Paniccia, Cristiana De Ranieri, Matilde Angeloni, Beatrice Arlini, Alessandro Capuano, Roberto Frusciante, Federico Vigevano, Simonetta Gentile, Massimiliano Valeriani

**Affiliations:** Unit of Neurology, Ospedale Pediatrico Bambino Gesù, IRCCS, Rome, Italy; Unit of Clinical Psychology, Ospedale Pediatrico Bambino Gesù, IRCCS, Rome, Italy; Center for Sensory-Motor Interaction, Aalborg University, Aalborg, Denmark

## Background

Migraine, epilepsy and tics are common neurological disorders in children and adolescents. They can affect a patient's life in a number of ways such as their school, sport and relationships. Although they are clearly different conditions, several studies have stressed the co-occurrence of migraine with both epilepsy and tic disorders. However, no study has compared the psychological/behavioural profile of children/adolescents with migraine, RAP or tics. The main aim of the present study was to compare the occurrence of internalizing and externalizing disorders between migraine, epilepsy and tics patients.

## Methods

We studied 32 migraine patients (m.a. 11.8 years; s.d 2.6; F: 19; M: 13), 25 epilepsy-normal IQ outcome (m.a. 15 years; s.d 2.6; F: 15; M: 10) and 29 tics (simple and multiple) (m.a. 8.8 years; s.d 2.6; F: 8; M: 21). The psychological profile was evaluated by the Child Behaviour Checklist 6-18 (CBCL). ANOVA one-way analysis was used to compare CBCL scales and subscales between groups.

## Results

Migraine, epilepsy and tics showed a very similar trend in the Internalizing scale (p = 0.12). Tics had higher scores in Externalizing (p = 0.00) and Total scores (p = 0.00). While “Anxiety/depression” and “Withdrawn” scores did not show any significant difference among the three groups (respectively, p = 0.06 and p = 0.72), migraineurs had a significant higher score in “Somatic complaints” subscale, compared with epilepsy (p = 0.00).

## Conclusions

Anxiety and depression are common psychological issues among children with migraine, epilepsy and tics. Moreover, our results suggest that although the three conditions did not show differences in internalizing symptoms, migraine tends to report higher levels of somatic complaints. On the other hand, tics are more prone to behavioural problems.

Written informed consent to publish was obtained from the patient(s).

